# Evaluation of QuantiFERON-TB Gold Plus for Detection of *Mycobacterium tuberculosis* infection in Japan

**DOI:** 10.1038/srep30617

**Published:** 2016-07-29

**Authors:** Lina Yi, Yuka Sasaki, Hideaki Nagai, Satoru Ishikawa, Mikio Takamori, Kentaro Sakashita, Takefumi Saito, Kiyoyasu Fukushima, Yuriko Igarashi, Akio Aono, Kinuyo Chikamatsu, Hiroyuki Yamada, Akiko Takaki, Toru Mori, Satoshi Mitarai

**Affiliations:** 1 Department of Mycobacterium Reference and Research, The Research Institute of Tuberculosis, Japan Anti-Tuberculosis Association, Kiyose, Japan; 2 Department of Respiratory Medicine, Fukujuji Hospital, Japan Anti-Tuberculosis Association, Kiyose, Japan; 3 Department of Basic Mycobacteriology, Graduate School of Biomedical Sciences, Nagasaki University, Nagasaki, Japan; 4 Department of Respiratory Medicine, National Hospital Organization Tokyo National Hospital, Kiyose, Japan; 5 Department of Respiratory Medicine, National Hospital Organization Chiba Higashi National Hospital, Chiba, Japan; 6 Department of Respiratory Medicine, Tokyo Metropolitan Tama Medical Centre, Fuchu, Japan; 7 Department of Respiratory Medicine, National Hospital Organization Ibaraki Higashi National Hospital, Ibaraki, Japan; 8 Department of Respiratory Medicine, Nagasaki Genbaku Isahaya Hospital, Nagasaki, Japan

## Abstract

Performance of interferon-γ (IFN-γ) release assays still needs to be improved. The data on the performance of QuantiFERON-TB Gold Plus (QFT-Plus), a new-generation of QFT assay are limited. This study evaluated the diagnostic performance of QFT-Plus, and compared to that of QuantiFERON-TB Gold In-Tube (QFT-GIT). Blood samples were collected from 162 bacteriologically confirmed tuberculosis (TB) patients and 212 *Mycobacterium tuberculosis*-uninfected volunteers; these samples were then tested with QFT-GIT and QFT-Plus. The IFN-γ concentration of QFT-Plus was lower than that of QFT-GIT in TB patients (p < 0.001). Receiver operating characteristic curves were compared between QFT-GIT and QFT-Plus. Both assays showed area under the curve values over 0.99 without significant difference. Using the conventional cut-off (0.35 IU/mL) for QFT-GIT, QFT-Plus had a lower sensitivity of 91.1% compared to 96.2% (p = 0.008) at its optimum cut-off (0.168 IU/mL) with the same specificity. Moreover, IFN-γ values were significantly reduced with age in QFT-GIT (p = 0.035) but not in QFT-Plus. The diagnostic performance of QFT-Plus was as accurate as that of QFT-GIT despite a lack of TB7.7 antigen and despite the decrease in quantitative values. However, the cut-off value for QFT-Plus should be considered independently from that of QFT-GIT to obtain the best sensitivity without compromising specificity.

Tuberculosis (TB) remains a serious health concern, and approximately one-third of the global population has been infected with its causal pathogen *Mycobacterium tuberculosis* (M.tb)^1^. The incidence of TB in Japan was estimated at 18 per 100,000 individuals in 2014^2^. The tuberculin skin test (TST) has been the primary approach to detect M.tb infection; however, due to antigenic cross-reactivity with Bacillus Calmette-Guérin (BCG) and the majority of non-tuberculosis mycobacteria, false-positive responses could occur^3^,^4^. The interferon (IFN)-γ release assay (IGRA) detects M.tb infection by measuring immunologic responses of T-cells, which release IFN-γ following stimulation by specific M.tb antigens^5^. Unlike TST, IGRA avoids the potential for cross-reaction with BCG vaccination^3^. Currently, two commercial IGRAs are available: QuantiFERON^®^TB Gold In-Tube (QFT-GIT; Qiagen, Germantown, USA) and T-SPOT.*TB* (Oxford Immunotec, Abingdon, UK). Both assays are whole-blood tests and are used for detecting infection with M.tb.

QFT-GIT assay measures IFN-γ concentration to detect M.tb infection using a single mixture of synthetic peptides representing ESAT-6, CFP-10, and TB7.7 (Rv2654c) to stimulate T-cells^5^. The ESAT-6 and CFP-10 antigens contained in QFT-GIT are epitopes for both CD4^+^ and CD8^+^ T cells^6^,^7^,^8^, but mainly stimulate CD4^+^ T cells to release IFN-γ. However, recent studies have indicated that both CD4^+^ and CD8^+^ antigen-specific T cells secrete cytokines, including IFN-γ^9^,^10^,^11^,^12^, when exposed to M.tb antigens. Since both CD4^+^ and CD8^+^ T cells can release IFN-γ after stimulation with M.tb antigens^13^,^14^,^15^, the addition of a CD8^+^ cytotoxic T-cell stimulating peptide may have the potential to improve the sensitivity in diagnosing infection with M.tb. Therefore, Qiagen Company developed the next generation of a QFT assay, QuantiFERON-TB Gold Plus (QFT-Plus), with new formulations of ESAT-6/CFP-10 peptides contained in two tubes, tube 1 (TB1) and tube 2 (TB2). TB1 contained relatively long synthetic peptide cocktails to mainly stimulate CD4^+^ T cells, whereas TB2 also contained short peptide cocktails to stimulate both CD4^+^ and CD8^+^ T cells^16^.

The pooled sensitivity and specificity of QFT-GIT for detecting confirmed active TB were reported by Diel *et al*.^17^, which were 0.81 and 0.99, and those of T-SPOT.*TB* were 0.88 and 0.86, respectively, suggesting that QFT-GIT has lower sensitivity than T-SPOT.*TB*, using a 6-spot cut-point and blood samples less than 8 hours old. Since there is no gold-standard for diagnosing latent M.tb infection (LTBI), we used patients with culture-positive TB and M.tb-uninfected healthy subjects as reference standards in this study. We assessed IFN-γ concentrations obtained from QFT-Plus assay in the culture-confirmed TB patients and in the healthy subjects. Then we compared the diagnostic accuracy of QFT-Plus with that of QFT-GIT at various cut-off values for diagnosing active TB in Japan. A cut-off value of 0.1 IU/mL, which is currently used in Japan as a second cut-off for individuals with a high probability of M.tb infection, was also evaluated in this study^18^.

## Methods

### Study Participants

**Figure 1:**
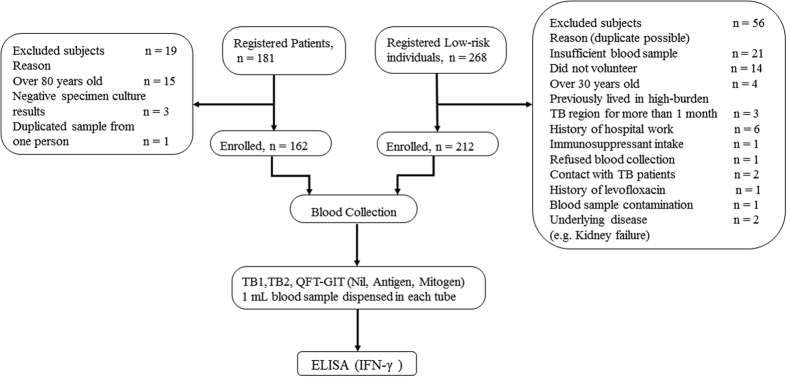
Flow diagram and study design. The reasons for exclusion of the registered patients and low-risk individuals are shown in Fig. 1. A 1 mL blood sample collected from enrolled TB patients and low-risk individuals was dispensed into each tube (TB1 and TB2 tubes of QFT-Plus assay, and nil, mitogen, antigen tubes of QFT-GIT assay). After incubation and centrifugation, the supernatant plasma of each tube was used to perform ELISA and to obtain the concentration of released IFN-γ. QFT-GIT: QuantiFERON-TB Gold In-Tube; ELISA: enzyme linked immunosorbent assay; TB1, TB2: Antigen tubes of QuantiFERON-TB Gold Plus.

We enrolled bacteriologically confirmed active TB patients as the positive controls, and healthy volunteers assumed to be M.tb-uninfected as negative standards to obtain the sensitivity in the TB patients and specificity in the healthy volunteers, respectively. Figure 1 depicts the flow diagram for the data collection and analyses in this study^19^.

### Patients with Active TB

Patients with bacteriologically confirmed (M.tb culture-positive) TB were enrolled from six hospitals (Fukujuji Hospital, Tokyo Hospital, Chiba Higashi Hospital, Ibaraki Higashi Hospital, Nagasaki Genbaku Isahaya Hospital, and Tama Medical Centre) between January 2014 and March 2015. Patients who had been treated with anti-TB drugs for more than two weeks were ineligible for this study. Patients were asked to provide information on their TB treatment history and other medications that may induce immunosuppressive conditions, including anticonvulsants, immunosuppressants, corticosteroids, anti-cancer chemotherapy, and any biological response modifier.

### Subjects Assumed to be M.tb-uninfected

Healthy subjects who were born in Japan and were less than 30 years old at the time of enrolment were considered to have no or a very low risk of M.tb infection, since the estimated prevalence of M.tb infection of this population is less than 1%^20^. All participants were volunteers, recruited from six institutes in the Tokyo metropolitan area and Saitama prefecture. The subjects were asked to complete a questionnaire indicating their date of birth, gender, residence history, BCG vaccination status, TST results (if available), history of contact with TB patients, any history of immunocompromised status, and any medication. Subjects who had lived in countries with a TB incidence rate over 50/100,000 for over one month, or who had worked in nursing homes or hospitals with a TB ward for over one month were ineligible for this study.

### Blood Sample Collection

A 10-mL whole blood sample was drawn in two blood-collecting vessels containing lithium-heparin. The blood samples were transported to the laboratory within 6 hours at controlled temperature (22 ± 5 °C), and a 1-mL sample was dispensed into each assay tube and incubated at 37 ± 1 °C for 16–24 h within 16 h after collection. Testing procedures for QFT-Plus and the conventional QFT-GIT require 5 tubes; i.e. TB1, TB2, the conventional antigen tube of QFT-GIT, and positive (mitogen) and negative (no antigen, nil) controls.

After incubation, the tubes were centrifuged at 2,330 × *g* for 15 min. A total of 300 μL of plasma was harvested and stored at −80 °C until performing the enzyme-linked immunosorbent assay (ELISA). The standard QFT ELISA kit was used for ELISA, the frozen plasma sample was thawed at room temperature and re-centrifuged at 1,750 × *g* for 15 min. Samples from 8 subjects were analysed on a single plate in each run of ELISA, which also included 4- and 8-point standards that were measured in duplicate. The optical density of each well was measured on a plate reader (Multiskan JX, Thermo Scientific) using the QFT-GIT analysis software (Ascent Software Version 2.6, Thermo Scientific). The concentration of released IFN-γ in each tube was calculated by subtracting the value of the nil (negative control) tube. If the coefficient of variation for the result was less than 15% and the correlation coefficient for the standard curve was greater than 0.98, the assay was considered to be technically valid. All of the results were interpreted by referring to a 4-point standard curve.

The ELISA results of the QFT-GIT and QFT-Plus test were interpreted as follows. A positive test was defined as antigen −nil ≥ 0.35 IU/mL and ≥ 25% of the nil sample, where as a negative test was defined as antigen–nil < 0.35 IU/mL or < 25% of nil, when mitogen ≥ 0.5 IU/mL. The results were considered indeterminate if 1) nil > 8 IU/mL or 2) antigen–nil ≥ 0.35 IU/mL and < 25% of nil when the nil was ≤ 8.0 IU/mL and the mitogen response was <0.5 IU/mL. Furthermore, a cut off of 0.1 IU/mL is a second cut-off for QFT-GIT used in Japan. The results are considered likely positive if 0.35 > antigen - nil ≥ 0.1, when mitogen ≥ 0.5 IU/mL. In such cases, the results need to be retested. The test results are considered negative if antigen - nil < 0.1, when mitogen ≥ 0.5 IU/mL. All of the final results were recorded on a spreadsheet.

### Statistical Analysis

Statistical Package for the Social Sciences (SPSS) version 22.0 and Medcalc version 15.10.0 for Windows (Chicago, IL, USA) were used for statistical analyses. The area under the curve (AUC) and cut-off values of each tube were obtained from the receiver operating characteristic (ROC) curve. The χ^2^ test was used to compare categorical variables between groups, as appropriate. The Mann Whitney test was used to analyse differences of IFN-γ values between groups. McNemar and Wilcoxon matched-pairs tests were used for comparing sensitivities and specificities, and IFN-γ values between different tubes, respectively. The trend in the change of IFN-γ values with age was estimated with the Jonckheere-Terpstra (JT) trend test. Excel 2013 (Microsoft, USA) was used to group data regarding age, gender, and information from the questionnaires. A p-value of < 0.05 was considered statistically significant.

### Ethical Considerations

This study was approved by the Institutional Ethical Committee of each hospital and the Research Institute of Tuberculosis. Written informed consent and completed questionnaires were obtained from each subject. The methods were carried out in accordance with the approved guidelines.

## Results

A total of 162 active TB patients and 212 subjects with a low risk of M.tb infection (low-risk subjects) were enrolled between January 2014 and March 2015. The median ages of the TB patients and low-risk subjects were 59 (interquartile range [IQR] of 39–70) and 20 (19–21) years, respectively (p < 0.001). A total of 129 patients (79.6%) and 105 low-risk subjects (49.5%) were male (p < 0.001). P values refer to the significance of differences in age and proportion of gender between the TB patients and the low-risk subjects. TB was bacteriologically confirmed in all patients, with noted complications of pleurisy (n = 5), tracheobronchial manifestations (n = 4), haematogenous disseminations (n = 2), and laryngeal diseases (n = 2). Three patients were on anticonvulsants, one was on an immunosuppressant, seven were taking corticosteroids, and one was receiving anti-cancer chemotherapy. A total of 25 (12%) low-risk subjects had not been BCG-vaccinated. The QFT-GIT test was positive (cut-off 0.35 IU/mL) in three low-risk subjects (1.4%) and in 147 of the patients (90.7%). None of the low-risk subjects (0%) but five patients (3.1%) showed indeterminate results (one case for nil > 8 IU/mL, and four cases for mitogen < 0.5 IU/mL and antigen–nil < 0.35 IU/mL). The characteristics of the study subjects and their clinical information are shown in Table 1.

**Figure 2:**
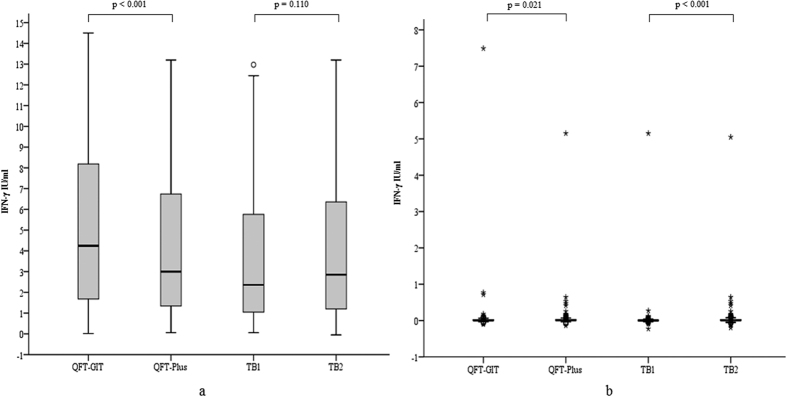
Interferon-γ concentrations of active TB patients (**a**) and low-risk subjects (**b**). The horizontal lines indicate the median. IFN-γ concentrations of QFT-GIT, QFT-Plus, TB1 and TB2 of TB patients and low-risk subjects are shown in Fig. 2a,b, respectively. The concentrations of IFN-γ released in QFT-GIT were significantly higher than those in QFT-Plus (p < 0.001). The concentrations of IFN-γ released in TB1 showed no significant difference compared to those obtained with TB2 (p = 0.110) in TB patients (Fig. 2a). QFT-GIT showed significantly lower IFN-γ concentrations compared to those obtained with QFT-Plus (p = 0.021) and TB1 showed significantly lower IFN-γ concentrations compared to those obtained with TB2 (p < 0.001) in low-risk subjects (Fig. 2b). The p-values shown were derived from Wilcoxon matched-pairs tests. A p value of < 0.05 was considered statistically significant. QFT-GIT: QuantiFERON-TB Gold In-Tube; QFT-Plus: QuantiFERON-TB Gold Plus; TB1, TB2; Antigen tubes of QFT-Plus.

The indeterminate cases were excluded from further statistical analyses. The median (IQR) amounts of released IFN-γ of each tube (TB1, TB2, QFT-Plus [calculated using the higher IFN-γ value from either TB1 or TB2 in each case], QFT-GIT) are shown in Table 2. The IFN-γ concentration of QFT-Plus was significantly lower than that of QFT-GIT in the patients (p < 0.001), whereas there were no significant differences between TB1 and TB2 (p = 0.110). In the low-risk subjects, QFT-GIT showed significantly lower IFN-γ concentrations compared to those obtained with QFT-Plus (p = 0.021), and TB1 showed significantly lower IFN-γ concentrations compared to those obtained with TB2 (p < 0.001) (Table 2, Fig. 2).

The diagnostic accuracy of each tube was analysed by ROC analyses. All of the AUC values of QFT-GIT, TB1, TB2, and QFT-Plus were greater than 0.98, and are listed in Table 3. The AUC values of QFT-Plus and QFT-GIT were not significantly different (p = 0.537). Based on Youden’s index, the optimal cut-off values are shown in Table 3. With these statistically derived cut-off values, the sensitivity and specificity were calculated and are shown in Table 3. The McNemar test showed that at each optimal cut-off value, there was no significant difference between the sensitivity of QFT-Plus and that of QFT-GIT, or between the sensitivity of TB1 and that of TB2. The sensitivity, specificity, positive likelihood ratio, and negative likelihood ratio of each tube with different cut-off values are shown in Table 4. The McNemar test showed that at the conventional cut-off value of 0.35 IU/mL, the sensitivity of QFT-GIT was not significantly different from that of QFT-Plus (p = 0.289). Furthermore, there was no significant difference in sensitivity between TB1 and TB2 (p = 0.125). There was no significant difference in the specificities between QFT-GIT and QFT-Plus (p = 0.625). With the cut-off value of 0.1 IU/mL used in Japan^18^, both sensitivity and specificity were not significantly different between QFT-GIT and QFT-Plus. With the conventional cut-off value of 0.35 IU/mL, QFT-Plus had a lower sensitivity (91.1%) than that obtained (96.2%) at its optimum cut-off of 0.168 IU/mL (p = 0.008), whereas there was no significant difference in specificity with the different cut-off values. The JT test indicated that IFN-γ values were significantly reduced with increased age (p = 0.035) in QFT-GIT but not in TB1, TB2, and QFT-Plus.

Among the total 157 patients analysed, the overall results did not change after exclusion of the eight patients receiving medications that may induce immunosuppression.

## Discussion

We evaluated the performance of a new IGRA, QFT-Plus, in the setting in Japan. With ROC analyses, the AUC values of TB1, TB2, and QFT-Plus did not show any significant differences from that of the conventional QFT-GIT. This finding indicates that the diagnostic performance of QFT-Plus was as accurate as that of QFT-GIT in this study.

The released IFN-γ values obtained with QFT-GIT for the patient samples were significantly higher than those obtained with the QFT-Plus system (Table 2, Fig. 2). Previous studies showed that the antigen TB7.7 was highly specific for TB^21^, and the amount of released IFN-γ was significantly higher in TB patients than in BCG-vaccinated controls^22^. QuantiFERON-TB Gold (QFT-G) was the second-generation QFT assay, which used only ESAT-6 and CPF-10 as antigens. A previous comparison of the performance of QFT-G and QFT-GIT showed that the IFN-γ response in the QFT-GIT test was significantly higher than that of the QFT-G test^23^. These studies may explain the observed differences in the amount of released IFN-γ between QFT-Plus and QFT-GIT in the present study, since QFT-Plus does not contain TB7.7, and thus may release less IFN-γ.

Although TB2 included modified peptides that are optimized to activate M.tb-specific CD8^+^ T cells, our results showed that in the TB patients, the secreted IFN-γ value in TB2 was similar to that in TB1. The first finding that IFN-γ was produced by CD8^+^ T cells was based on a study of CD4^+^ T cell-deficient mice^24^. Another study showed that mycobacteria-specific CD8^+^ T cells could produce IFN-γ without help from CD4^+^ T-cells^5^. Therefore, adding CD8^+^ T cell-stimulating antigens may increase the amount of IFN-γ released, compared to the case in which only CD4^+^ T cell-stimulating antigens are present. Furthermore, CD8^+^ T cells preferentially recognise cells from a host heavily infected with M.tb, and the response from CD8^+^ T cells correlates well with the bacterial load^25^. Moreover, the response from M.tb-specific CD8^+^ T cells was reported to decrease by 58.4% at 24 weeks after anti-TB treatment^26^. Since the TB patients enrolled in our study had not received any TB treatment for more than two weeks prior to testing, the secreted IFN-γ value in TB2 should have been higher than that in TB1. However, there was no significant difference between the IFN-γ values in TB1 and TB2 for the patients in this cohort, inferring that IFN-γ release was not amplified by the CD8^+^ T cell-stimulating antigens. This finding differs from what was published recently^16^. However, a review of their results suggests that a high percentage of smear-positive patients with high bacterial load in their series. In fact, among 15 cases with smear results of more than 10 acid-fast bacilli per 100 fields in our study, the difference in the quantitative response of TB2 compared to that of TB1 almost reached significance (p=0.053). This may explain the discrepancy of these two studies. A previous study indicated that during M.tb infection, IFN-γ production is mostly derived from CD4^+^ T cells, with a smaller contribution from CD8^+^ T cells^27^. This may explain why the amount of IFN-γ released did not substantially vary between TB1 and TB2 in the present study.

Harada *et al*.^23^ demonstrated that the sensitivity and specificity of QFT-GIT were 92.6% and 98.8%, respectively, using the standard cut-off of 0.35 IU/mL in Japan. These results are similar to our results for QFT-GIT with the same cut-off (sensitivity: 93.6%; specificity: 98.6%). The McNemar test showed that at the cut-off value of 0.35 IU/mL, the sensitivities and specificities of QFT-GIT and QFT-Plus were similar. Nevertheless, given that QFT-GIT and QFT-Plus are different diagnostic systems, their performance should be evaluated independently.

The sensitivities and specificities of IGRAs have been shown to vary in TB patients with HIV co-infection and in children^28^,^29^. In young children, IFN-γ-producing M.tb-specific CD8^+^ T cells respond to high loads of M.tb following TB exposure^30^. QFT-Plus was developed based on these observations of protective CD8^+^ T cell responses, with the aim to improve the sensitivity of the assay for children and immunocompromised individuals with low CD4^+^ T cell counts. However, TB2 did not show a higher sensitivity or specificity than TB1 (Table 3). We suspect that this may be because most of the subjects enrolled in this study had intact immunity. The mitogen value of one excluded patient who was taking a corticosteroid was 0.45 IU/mL, indicating immune-deficient status of this patient. The amount of IFN-γ released in TB2 in this case was 0.7 IU/mL, which was higher than that of TB1 (0.21 IU/mL). This may indicate a specific effect of the release of IFN-γ from CD8^+^ T cells. Further studies targeting immunocompromised subjects may confirm the value of the TB2 assay in such patients.

In the present study, the IFN-γ levels obtained with QFT-Plus were lower than those obtained with QFT-GIT. In our study, the statistically derived cut-off value of QFT-Plus used for the accuracy analysis was 0.168 IU/mL, which is much lower than the currently recommended value of 0.35 IU/mL; thus, the cut-off value of QFT-Plus may be set to be lower than the current standard of 0.35 IU/mL. QFT-Plus showed significantly lower sensitivity (91.1%) calculated at the conventional cut-off value of 0.35 IU/mL when compared to its sensitivity (96.2%) calculated at the optimum cut-off value of 0.168 IU/mL (p = 0.008). This indicated that the cut-off for QFT-Plus should be reconsidered independently from that of QFT-GIT. We evaluated the diagnostic performance using different cut-off values (Table 4), and, as expected, found a trade-off relationship in which the specificity increased but sensitivity decreased by raising the cut-off values. In particular, when the cut-off was set to 0.35 IU/mL, the specificity of TB1 increased to nearly 100%. Because of this variation, as a matter of medical practice, diagnosis of M.tb infection should be based on not only interpretation of the cut-off value but also on epidemiological and/or clinical assessment^31^.

The CD8^+^ T cell response was reported to be enhanced in subjects who had recent contact with TB patients, whereas the CD4^+^ T cell response seemed to correlate with active TB in patients tested with QFT-GIT^32^. Thus, the CD8^+^ T cell response may be stronger at the onset of infection, as observed in a bovine model of M.tb infection^33^. However, our current study may be insufficient for the direct assessment of the response of CD8^+^ T cells, since we did not include patients with immunodeficiency and young children, and did not evaluate the individual time course of each patient from exposure to having active TB. In addition, we could not classify the patients according to background factors such as severity of TB and ethnicity, owing to the lack of information. Further studies are needed to investigate the utilities of QFT-Plus by classifying the background factors. Such information may help to identify the time course of M.tb infection as well as the treatment effect. Although QFT assay was developed to detect M.tb infection, in the current study we evaluated this assay using active TB patients only. Further studies should focus on not only active TB patients but also close contacts of TB patients who have a high possibility of asymptomatic M.tb infection in order to obtain data evaluating the performance of this new QFT assay. The diagnostic performance of our study should only be considered in the setting like Japan, where has a population with very low level of LTBI^2^, but not in typically high-TB settings and/or high HIV-burden settings as WHO indicated^34^.

In conclusion, we confirmed that the performance of QFT-Plus is as accurate as that of QFT-GIT despite a lack of TB7.7 antigen and despite decrease in quantitative values for individuals with intact immunity. Since QFT-Plus has the potential ability to detect M.tb infection in immunodeficient individuals including HIV-positive patients and small children, QFT-Plus could replace the current QFT-GIT assay in Japan. The cut-off value of QFT-Plus should be considered independently from the conventional cut-off for the new modality. However, since QFT is mainly used as a test for LTBI and not for active TB, the optimized cut-off value found in this study cannot be generalized as the standard for LTBI screening. Further study is warranted to confirm the optimized cut-off value.

## Additional Information

**How to cite this article**: Yi, L. *et al*. Evaluation of QuantiFERON-TB Gold Plus for Detection of *Mycobacterium tuberculosis* infection in Japan. *Sci. Rep.* **6**, 30617; doi: 10.1038/srep30617 (2016).

## Figures and Tables

**Table 1:** Clinical and demographic characteristics of patients and low-risk subjects.

	Patients (n = 162)	Low-risk subjects (n = 212)	p value
Age (y), median (IQR)	59 (39–70)	20 (19–21)	p < 0.001*
Male, n (%)	129 (79.6%)	105 (49.5%)	p < 0.001**
Smear results (n)	positive (138)	NA	NA
	negative (24)		
Type of TB (n)	Pulmonary TB (162)	NA	NA
	Tuberculosis pleurisy (5)		
	Tracheobronchial TB (4)		
	Miliary TB (2)		
	Laryngeal TB (2)		
Concomitant Drugs (n)	Anticonvulsant (3)	NA	NA
	Corticosteroid (5)		
	Immunosuppressant and Corticosteroid (1)		
	Corticosteroid and Cancer Chemotherapy (1)		
	Monoclonal Antibody (0)		
History of BCG vaccine (n)	Unknown	yes (187)	NA
		uncertain (25)	

^*^Mann-Whitney *U*-test.

^**^Chi-square test.

Low-risk subjects refer to the subjects with a low risk of M.tb infection. Data of age are expressed as the median (IQR). P values refer to the significance of differences in age and proportion of gender between the TB patients and low-risk subjects. QFT-GIT: QuantiFERON-TB Gold In-Tube; TB: tuberculosis; BCG: Bacillus Calmette-Guérin; IQR: interquartile range; NA: not applicable.

**Table 2:** Concentration of IFN-γ (IU/mL) measured using the different antigen-containing tubes in patients and in low-risk subjects.

Tube No.	Patients	Low–risk subjects
TB1	2.359 (1.040–5.840)	0.003 (−0.006–0.012)
TB2	2.850 (1.147–6.365)	0.009 (−0.003–0.029)
QFT-Plus	3.000 (1.280–6.753)	0.010 (0.000–0.030)
QFT-GIT	4.243 (1.668–8.225)	0.007 (0.000–0.026)

Data are expressed as the median (IQR). TB1 and TB2 are antigen-containing tubes of QFT-Plus. The QFT-Plus assay results were calculated using the higher IFN-γ value from either TB1 or TB2 in each case. QFT–GIT: QuantiFERON-TB Gold In-Tube; QFT-Plus: QuantiFERON-TB Gold Plus. IQR: interquartile range.

**Table 3:** Different parameters calculated using the different antigen-containing tubes.

Tube No.	AUC (95% CI)	Std. Error	Cut-off point	Sensitivity (%) (95% CI)	Specificity (%) (95% CI)	LR+ (95% CI)	LR– (95% CI)
TB1	0.995 (0.988–1.000)	0.003	0.085	99.4 (96.5–100)	97.6 (94.6–99.2)	42.1 (17.7–100.2)	0.01 (0–0.05)
TB2	0.987 (0.973–1.000)	0.004	0.168	96.2 (91.9–98.6)	96.7 (93.3–98.7)	29.1 (14.1–60.4)	0.04 (0.02–0.09)
QFT-Plus	0.992 (0.995–0.999)	0.004	0.168	96.2 (91.9–98.6)	96.7 (95.9–99.7)	29.1 (22.1–209.1)	0.04 (0.02–0.08)
QFT-GIT	0.990 (0.981–0.999)	0.005	0.196	96.2 (91.9–98.6)	98.6 (95.9–99.7)	68 (22.1–209.1)	0.04 (0.02–0.08)

The optimised cut-off point of each tube was calculated from the receiver operating characteristic analysis. Using the optimised cut-off point, sensitivity, specificity, LR+ and LR- of each tube were calculated. TB1 and TB2 are antigen-containing tubes of QFT-Plus. There was no significant difference between AUCs calculated from QFT-Plus and QFT-GIT (p = 0.537). AUC: area under curve; QFT-GIT: QuantiFERON-TB Gold In-Tube; QFT-Plus: QuantiFERON-TB Gold Plus; LR+: positive likelihood ratio; LR-: negative likelihood ratio. CI: confidence interval.

**Table 4:** Diagnostic performance of QFT-Plus and QFT-GIT for detection of active TB using different cut-off points.

Cut-off point (IU/mL)	Tube No.	Sensitivity (%) (95% CI)	Specificity (%) (95% CI)	LR+ (95% CI)	LR– (95% CI)
0.1	TB1	97.5 (93.6–99.3)	98.1 (95.2–99.5)	51.7 (19.6–136.4)	0.03 (0.01–0.07)
	TB2	99.4 (96.5–100)	90.6 (85.8–94.1)	10.5 (6.9–16.0)	0.01 (0.00–0.05)
	QFT-Plus	99.4 (96.5–100)	90.6 (85.8–94.1)	10.5 (6.9–16.0)	0.01 (0.00–0.05)
	QFT-GIT	96.2 (91.9–98.6)	95.3 (91.5–97.7)	20.4 (11.1–37.4)	0.04 (0.02–0.09)
0.35	TB1	88.5 (82.5–93.1)	99.5 (97.4–100)	187.7 (26.5–1327.4)	0.12 (0.07–0.18)
	TB2	91.1 (85.5–95.0)	97.6 (94.6–99.2)	38.6 (16.2–92.0)	0.09 (0.06–0.15)
	QFT-Plus	91.1 (85.5–95.0)	97.6 (94.6–99.2)	38.6 (16.2–92.0)	0.09 (0.06–0.15)
	QFT-GIT	93.6 (88.6–96.9)	98.6 (95.9–99.7)	66.2 (21.5–203.7)	0.06 (0.04–0.12)

Sensitivity, specificity, LR+, LR- were calculated in each tube using cut-off point 0.1 IU/ml or 0.35 IU/ml, respectively. LR+: positive likelihood ratio; LR-: negative likelihood ratio; QFT-GIT: QuantiFERON-TB Gold In-Tube; QFT-Plus: QuantiFERON-TB Gold Plus.
